# Investigating omega-3 fatty acids’ neuroprotective effects in repetitive subconcussive neural injury: Study protocol for a randomized placebo-controlled trial

**DOI:** 10.1371/journal.pone.0321808

**Published:** 2025-04-24

**Authors:** Lauren H. Beauregard, Jeffrey J. Bazarian, Blair D. Johnson, Hu Cheng, Gage Ellis, William Kronenberger, Philip C. Calder, Zhongxue Chen, Patricia Silveyra, Patrick D. Quinn, Sharlene D. Newman, Timothy D. Mickleborough, Keisuke Kawata

**Affiliations:** 1 Department of Kinesiology, Indiana University School of Public Health-Bloomington, Bloomington, Indiana, United States of America; 2 Department of Emergency Medicine, University of Rochester School of Medicine and Dentistry, Rochester, New York, United States of America; 3 Program in Neuroscience, The College of Arts and Sciences, Indiana University, Bloomington, Indiana, United States of America; 4 Department of Psychological and Brain Sciences, College of Arts and Sciences, Indiana University, Bloomington, Indiana, United States of America; 5 Department of Psychiatry, Indiana University School of Medicine, Indianapolis, Indiana, United States of America; 6 School of Human Development and Health, Faculty of Medicine, University of Southampton, Southampton, United Kingdom; 7 NIHR Southampton Biomedical Research Centre, University Hospital Southampton, Southampton, United Kingdom; 8 College of Health Solutions, Arizona State University, Phoenix, Arizona, United States of America; 9 Department of Environmental and Occupational Health, School of Public Health-Bloomington, Indiana University, Bloomington, Indiana, United States of America; 10 Department of Applied Health Science, School of Public Health-Bloomington, Indiana University, Bloomington, Indiana, United States of America; 11 Alabama Life Research Institute, University of Alabama, Tuscaloosa, Alabama, United States of America; PLOS: Public Library of Science, UNITED KINGDOM OF GREAT BRITAIN AND NORTHERN IRELAND

## Abstract

Soccer (football) is the most popular sport globally, with 265 million players across all ages and sexes. Repetitive subconcussive head impacts due to heading of the soccer ball can pose threats to healthy brain development and aging. Omega-3 fatty acids, especially docosahexaenoic acid (DHA) and eicosapentaenoic acid (EPA), may have neuroprotective effects, but it remains unclear what aspects of neural health benefit from DHA+EPA when faced with subconcussive head impacts. In a randomized placebo-controlled trial, 208 soccer players will complete baseline measures including demographics, blood sampling, dietary recalls, and psychological assessment. Participants will be randomly assigned to ingest DHA+EPA [3.4g/d: DHA 2.4g+EPA 1.0g] or placebo daily for 8 weeks followed by a subconcussion intervention phase. During the subconcussion intervention, participants will perform a session of 20 controlled soccer headings, with a second session 24 hours later. Blood samples, neuroimaging data, autonomic reactivity, and clinical measures (symptoms, oculomotor, cognition) will be collected pre-heading and 24-hour post-1^st^ session, 24-hour post-2^nd^ session, and 7-day post-2^nd^ session. The primary hypothesis is that DHA+EPA pretreatment will promote neuronal and astrocyte resiliency to subconcussive head impacts, as assessed by blood biomarkers of brain injury, axonal microstructure measured by diffusion tensor imaging, and whole-brain resting-state connectivity. It is proposed that pretreatment will preserve autonomic function, as assessed by the cold pressor test (CPT), as well as oculomotor and cognitive function, even after head impacts. Data from this trial will help clarify the combined effect of DHA+EPA on brain molecular, cellular, and physiological health in response to subconcussive head impacts. If the hypotheses are confirmed, the findings will support a highly practical intervention for mitigating the neurodegenerative cascade triggered by head impacts.

**Trial registration:** ClinicalTrials.gov NCT06736925

## Introduction

Subconcussive head impacts are incredibly common among soccer players due to heading of the soccer ball, and their potential long-term effects have been highlighted in recent studies [1,2]. Former professional soccer players were found to have a mortality rate from neurodegenerative disease [especially Alzheimer disease (AD)] 5 times that of controls and were 4 times as likely to receive dementia-related medications [1]. These risks were modulated by career length, and positions with high exposure to headings (defenders, midfielders) [3,4] were more affected [2]. Repetitive head impact can also induce acute changes in brain cellular and functional integrities, where soccer headings, American football tackles, and boxing have been shown to elevate circulating tau, NF-L, UCH-L1, GFAP, and S100B levels, indicative of neural/glial injury and inflammation [5–12], as well as impairments in neuro-ophthalmologic (e.g., convergence, saccades) [13–16] and vestibular functions [17]. Although policy/rule changes to minimize head impact exposure are a logical step, subconcussive head impact inherent to athletic drills are difficult to regulate (e.g., soccer heading, tackles in contact sports). It is vital to explore and rigorously characterize potential interventions to enhance brain resiliency to subconcussive head impacts.

### Prophylactic and therapeutic effect of omega-3 fatty acids (FAs) on neural damage

#### Preclinical evidence.

DHA and EPA and long chain omega-3 fatty acids (FAs); they comprise 97% of brain omega-3 FAs (mostly DHA), are enriched in neuronal plasma phospholipid membranes and promote neurite outgrowth [18]. Studies have suggested that DHA and EPA exert potent protective effects against experimental traumatic brain injury (TBI). For example, the importance of DHA in maintaining brain resiliency to TBI was noted by Desai et al.[19] such that after sustaining a controlled TBI, DHA depleted mice (70% loss of DHA in the brain due to dietary manipulation) exhibited slower recovery from motor deficits, greater anxiety-like behavior and cognitive deficits, and elevated levels of alpha spectrin II breakdown products (axonal injury markers) compared to DHA adequate mice [19]. Mills et al.[20] further demonstrated that compared to untreated rats, rats pretreated with DHA showed significant reductions in anatomical damage after TBI, as measured by β-amyloid precursor protein counts within injured axons, axonal bulbs, and enhancement of cell survival (↓caspase-3). The effects of DHA on these cellular responses translated into cognitive resiliency, where rats pretreated with DHA showed similar memory function after TBI compared to sham rats, whereas untreated rats showed significant cognitive declines after TBI [21,22].

#### Clinical evidence.

Clinical evidence supports the potential of DHA and EPA to mitigate neuronal injury and mental illness, reduce blood pressure [23–26], attenuate sympathetic hyperreactivity [27], and enhance autonomic-regulated gut microbiota [28]. Bailes et al.[29] reported a case series of 9 comatose patients with severe TBI who were supplemented with a high dose of combined DHA+EPA (16.2g/d). All patients’ GCS scores improved over time, and they showed a significant return of cognitive and physical functions. The studies by Heileson et al.[30] and Oliver et al.[31] supplemented college football players daily with 2g DHA+0.56g EPA or placebo and with 2g, 4g, or 6g of DHA or placebo, respectively, and examined serum NF-L (indicative of axonal injury) changes across a season. The DHA+EPA group maintained NF-L levels throughout the season, whereas the placebo group showed significant elevations in NF-L levels [30]. Especially the players who ingested 2g/d DHA benefited the most, by maintaining NF-L levels similar to baseline throughout the season, whereas the players on placebo showed 60%-120% increases in NF-L levels [31]. However, because the studies did not record number or magnitude of head impacts and failed to account for various extraneous variables (e.g., exercise, body damage), the mechanism underlying the effect of DHA+EPA against subconcussive head impact remain speculative. The proposed study will address this question using a heading model and multimodal assessments.

### Aims and hypothesis

The proposed study will define the role and effectiveness of DHA+EPA in enhancing neural health and resiliency to acute subconcussive head impacts.

### Specific aims

- Aim 1: To test the hypothesis that DHA+EPA will promote neuronal and astrocyte resiliency to subconcussive head impacts, as assessed by a panel of blood biomarkers (NF-L, tau, GFAP, UCH-L1, S100B).
- Aim 2: To test the hypothesis that DHA+EPA will maintain axonal microstructure and functional connectivity after acute and cumulative soccer headings.
- Aim 3: To test the hypothesis that DHA+EPA will maintain normal sympathetic hyperreactivity after soccer headings, as reflected by a typical cardiovascular response to the CPT (cold-pressor test).
- Exploratory Aim 4: To explore the role of DHA+EPA in sustenance of neuro-ophthalmologic and cognitive functions following subconcussive head impacts.
- Exploratory Aim 5: To explore the interaction between attention-deficit/hyperactivity disorder (ADHD) and DHA+EPA, in response to subconcussive head impacts.

### Outcome measures

Our primary outcomes (Table 1) will be assessed by comparing group differences, specifically group (DHA+EPA vs placebo) x time interaction, at 24-hour post heading relative to pre-heading baseline. Secondary outcome measures (Table 1) will be assessed beyond 24 hours post heading, including 24 hours post 2nd heading session, and up to 7 days after 2^nd^ heading session.

**Table 1:** Outcome Measures.

Outcome Measure	Type	Test/Task and Description
Blood Biomarkers	Primary Outcome	The primary outcome analyses will be comparing group differences (group x time interactions) in blood biomarkers, specifically NF-L (neurofilament light), tau (picogram per milliliter), GFAP (glial fibrillary acidic protein; nanograms per milliliter), UCH-L1 (ubiquitin C-terminal hydrolase-L1; picograms per milliliter), and S100B (S100 calcium binding protein B; nanograms per milliliter).
Diffusion Tensor Imaging (DTI)	Primary Outcome	DTI will be used to derive mean diffusivity (MD; square millimeters per second) and fractional anisotropy (FA; a unitless value that ranges from 0 to 1, 0 = Isotropic, 1= Anisotropic) as indicators joint of axonal integrity (axonal microstructure) and connectivity of white matter tracts by measuring how water molecules move through brain tissues.
Sympathetic Reactivity	Primary Outcome	Sympathetic Reactivity will be measured by the Cold Pressor Test (CPT). CPT is a general assessment of the ability of the sympathetic nervous system to become activated. The main metric will be changes in mean arterial pressure (MAP) following soccer headings. This test will be performed by submerging a participant’s hand into cold water for 2 minutes while autonomic and hemodynamic variables are recorded. At each data collection, participants will be instrumented for the measurement of heart rate (electrocardiogram) and continuous blood pressure. Participants will rest quietly for ~10 minutes before the CPT begins. The test takes 2 minutes.
Oculo-Motor Function	Primary Outcome	Oculomotor function will be tested by Near point convergence (NPC) and the King-Devick test (KDT). NPC will be conducted Using the accommodative ruler, a target (14-point letter) will be moved toward the eyes at a rate of 1–2 cm/s. NPC will be recorded when participants report diplopia has occurred, or the tester observes eye misalignment. The assessment will be repeated twice, and the mean near point convergence value will be used for analyses. The King-Devick test (KDT) consists of a total of 145 saccades while rapidly reading numbers aloud to complete the test. The KDT will be administered on a tablet. The total time (in seconds) will be used for analysis.
Neurite orientation dispersion and density imaging (NODDI)	Secondary Outcome	NODDI metrics will be derived from the the diffusion tensor images using the NODDI toolbox v1.0 in Matlab. Maps of neurite density (ND), orientation dispersion (OD), and intracellular volume fraction (ICVF) will be generated.
Resting-state Functional Connectivity	Secondary Outcome	Resting-state connectivity will be examined throughout the whole brain and specific seeded regions including the dorsolateral prefrontal cortex (DLPFC), angular gyrus, and cingulate gyrus.
Cardiovagal Baroreflex Sensitivity (cBRS)	Secondary Outcome	Spontaneous cardiovagal baroreflex sensitivity (cBRS) will be collected over the last 5 minutes before the CPT. Baroreflex sequences of 4 consecutive cardiac cycles will be captured using WinCPRS software, for which directional changes in the R-R interval and corresponding systolic blood pressure will be identified. Sequences will be detected when changes in systolic blood pressure are ≥1mmHg and the variation in R-R interval is ≥5miliseconds. Only sequences with an R2 ≥0.85 will be deemed acceptable. The mean of the regression slope will be calculated as the cBRS gain and used for analysis
Heart Rate Variability (HRV)	Secondary Outcome	Leading up to and during the cold pressor test (CPT) heart rate will be monitored via 3-lead ECG. Resting-state heart rate variability and spontaneous cardiovagal baroreflex sensitivity (cBRS) will be monitored over the last 5 minutes before the CPT. Using the R-R interval data collected from the ECG recordings during 5 minutes of paced breathing, time domain analyses will be performed to estimate overall heart rate variability and provide insight into cardiac parasympathetic activity.
Cognition	Secondary Outcome	Cognitive function will be measured using the NIH Toolbox Cognition Battery, which has excellent reliability and validity to measure cognition in young adults and has construct validity among those with TBI. Multiple forms will minimize learning effects. This battery examines 5 cognitive domains (executive function, episodic memory, language, working memory, and processing speed) and takes 20 minutes to complete on a tablet.
Cerebral blood flow (CBF)	Secondary Outcome	Perfusion imaging will be acquired to examine cerebral blood flow.
Metabolomics	Secondary Outcome	Mitochondrial respiration related metabolites, including pyruvate, acetyl-CoA, citrate, isocitrate, alpha-ketogluterate, succinyl-CoA, succinate, fumarate, malate, and oxaloacetate will be assessed on blood samples. The aggregation of all metabolomic data will provide a comprehensive overview of the homeostasis of the participant following repetitive head injury.
Quantitative Susceptibility Mapping (QSM)	Secondary Outcome	Quantitative Susceptibility Mapping of whole brain, as well as regions of interest analysis, will be conducted to inspect the tissue and brain architectural response to head impacts and omega-3 fatty acid supplementation.
Genetic Markers	Exploratory Outcome	Several genetic markers that are known to associate with brain vulnerability will be assessed and regressed against our outcome measures. These genetic markers include, but not limited to, DARC, APOE, KIAA0319, BDNF, TPH2, COMT. Each marker will be compared to the other outcome measures to understand the full effect of the intervention on the genetic markers as a whole.
ADHD	Exploratory Outcome	Participants who meet criteria for ADHD diagnosis will be analyzed to address the interactive effects of DHA+EPA and head impacts in ADHD.

## Methods

### Subjects

This study will include 208 participants, who are between 18 and 30 years old and current or former soccer players with a minimum of five years of soccer heading experience. The recruitment has started since February 2025 until March 2028, and all data collection is expected to finish by July 2028. Primary analyses are anticipated to finish by June 2029.

Participants will be screened using the inclusion and exclusion criteria in Table 2. A participant may be withdrawn from the research without his/her consent if, they come to any session with intoxication, sustained head injury (e.g., concussion) during the study duration, exhibit any sign or action of violent behavior or verbal abuse toward research personnel. The Indiana University’s Institutional Review Board has approved the study protocol (#21334) on December 23, 2023.

**Table 2:** Inclusion and exclusion criteria.

Inclusion Criteria
18–30 years of age Current or former member of a collegiate, club, or professional soccer team Have 5+ years of soccer heading experience
**Exclusion Criteria**
Are currently pregnant Have had a head or neck injury within the past 6 months Have implanted metal/magnetic devices (e.g., orthodontic braces) Diagnosed with autonomic or cardiovascular disease (e.g., hypertension) Allergy to fish or shellfish Consuming omega-3 FA supplements including plant-based (e.g., flaxseed) in the past 3 months Consume oily fish (>2 servings/month: salmon, bluefin, swordfish, anchovies)

### Protocol proposal

#### Trial design.

**Fig 1:**
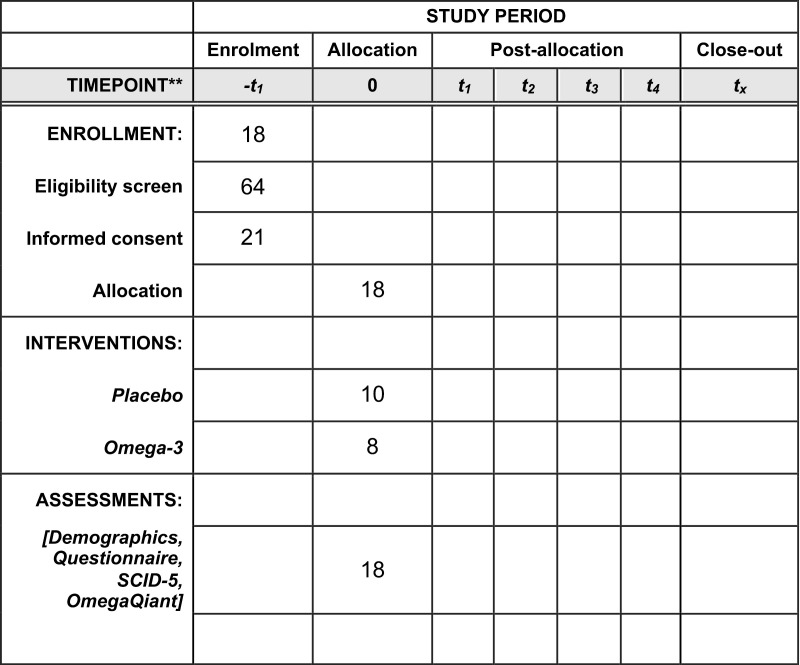
SPIRIT Checklist. SPIRIT schedule of enrollment includes participants who have been enrolled in the trial. Given the early stage of the study, no participants have advanced to outcome data collection as of March 2025.

**Fig 2:**
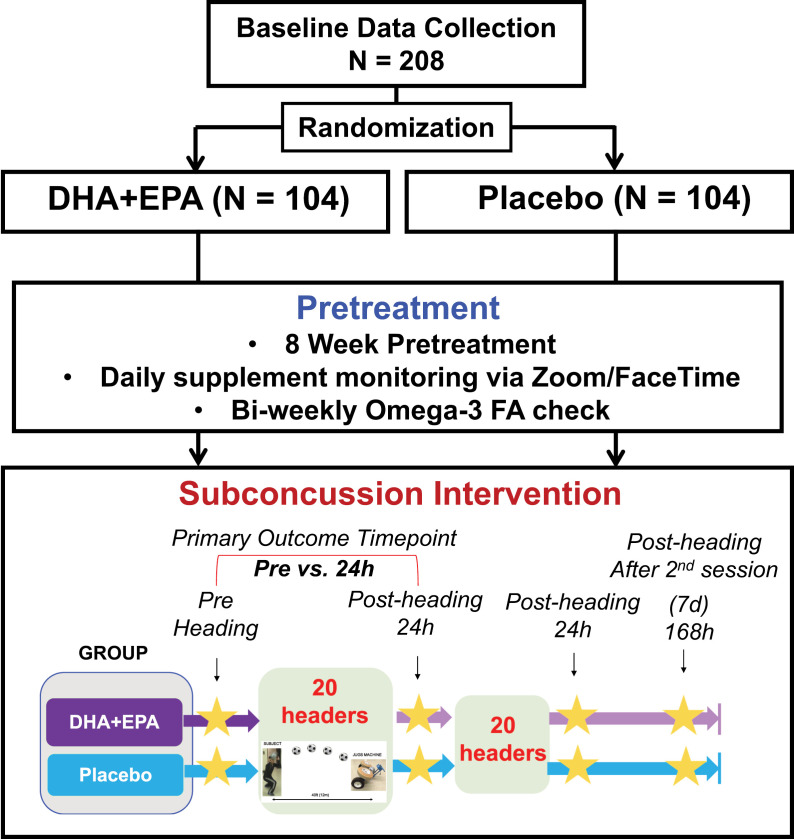
Schematic study design. Study figure depicts the flow of the study.

This randomized placebo-controlled trial will consist of 2 groups: (1) DHA+EPA and (2) Placebo (Fig 2). Written informed consent will be obtained from all participants by the study coordinators. If individuals meet inclusion/exclusion criteria, pre-supplement baseline data will be gathered, including demographics, blood spot sample for assessment of omega-3 FA composition, dietary recalls for estimation of antioxidant intake, and symptom checklist. Participants will ingest DHA+EPA or placebo supplements daily throughout the 8-week supplementation phase and the subconcussion intervention phase. Participants in both groups will be required to ingest the supplement while monitored virtually, via Zoom or Facetime. Female participants’ menstrual cycle (MC), as determined by self-report, which will be further validated by salivary progesterone levels, will be monitored during the supplementation phase to determine regular or irregular MC and MC phases. During the biweekly visit, a blood spot sample will be collected to measure DHA+EPA levels and other FAs in the blood. During the subconcussion intervention phase, participants will be fitted with a wrist-worn Actigraph to monitor their activity/sleep levels. The subconcussion intervention will involve performing 20 controlled soccer headings per session. Participants will undergo 2 sessions in a 2-day period. Blood sample, MR data, autonomic reactivity, and clinical measures (symptoms, oculomotor, cognition) will be collected at pre-heading and 24-hour post-1^st^ session, 24-hour post-2^nd^ session, and 7-day post-2^nd^ session. Fig 1 indicates the current status of enrollment and Fig 2 depicts the study flow.

### Randomization and blinding

Using the *R* randomization function, randomly stratified IDs that correspond to study groups will be generated. Randomly stratified sequentially numbered IDs will be placed in sealed envelopes by a blinded statistician, and containers of identical appearance will be prepared by a study coordinator, who will oversee the allocation of correct supplementation. Participants will be blinded to their group assignment. For participants with a vegetarian diet, they will be nonrandomly assigned equally to each group.

### Study interventions

#### DHA+EPA intervention.

Participants will receive a batch of supplements to last 2 weeks during bi-weekly visits and be instructed to ingest supplements during 1-min Zoom or FaceTime call with the research coordinator each day. For the DHA+EPA intervention, each soft gel capsule contains 480 mg of DHA, 205 mg of EPA, 145 mg of other omega-3 FAs, and 10 mg of vitamin E. Participants in the DHA+EPA group will ingest 5 capsules daily [a total of 3.4 g/d: DHA (2.4 g), EPA (1.0 g)]. Individual percentage changes in DHA+EPA composition at 8 weeks may be considered as a covariate in analyses if we observe heterogeneous changes in DHA+EPA levels. Organic soybean oil softgel capsules will be used as a placebo condition for the DHA+EPA capsules. One capsule contains 485 mg of organic soybean oil, including negligible amounts of DHA (1.1 mg), EPA (1.7 mg), and vitamin E (10 mg). Participants will ingest 5 capsules daily. The placebo capsule’s shelf life, composition, shape, and size are the same as the DHA+EPA capsules.

#### Blood fatty acid composition.

At baseline and after 8 weeks of supplementation, a fasting capillary blood sample from a fingertip will be collected and sent to OmegaQuant Analytics for blood FA composition analysis [32].

### Subconcussion intervention

A standardized and reliable soccer heading protocol will be used for the experiment. The ACT Head Impact Tracker head sensor Pro, embedded in a head-band pocket and positioned directly below the external occipital protuberance (inion) will be used to monitor linear and angular head accelerations. A JUGS soccer machine will be used to simulate a soccer throw-in with a standardized ball speed of 30 mph. Subjects will stand approximately 40 ft away from the machine to perform the heading [33]. Participants perform 20 headers with 1 header per 30 seconds. Both groups will undergo additional heading sessions 24 hours after the first session. The participants will be instructed to direct the ball in the air back toward the research staff standing 20ft in front of the participant. If the participant fails to return the ball in the air and/or within 60° in front, which will result in a low head acceleration, they will re-do the header.

### Measurements

#### Baseline measures.

The following information will be assessed at baseline: SCID-5-RV [34] for psychiatric diagnosis, blood spot for assessment of omega-3 FA compositions [32], dietary recall for estimation of antioxidant levels [35], clinical measures, demographic questionnaire, and medical and concussion history questionnaires. Participants will also complete the alcohol use disorders identification test (AUDIT) [36], Cannabis use disorder identification test – Revised (CUDIT-R),[37] Beck anxiety inventory (BAI) [38], General Anxiety Disorder-7 (GAD-7) [39], patient health questionnaire-9 (PHQ-9) [40–42], PTSD checklist – Civilian (PCL-C), Pittsburg sleep quality index (PSQI), DSM-5 Adult ADHD Checklist – Self-report [43], daily Stress inventory (DSI), the migraine disability assessment test (MIDAS), and Headcount-12 months.[44–46] Participants who screen positive for DSM-5 ADHD checklist will undergo the Adult ADHD Investigator Symptom Rating Scale (AISRS).

### Symptom checklist

At each timepoint, the post-concussion symptom scale, as a subset of the Sports Concussion Assessment Tool 5 [47], will be used to assess the presence and severity of concussion symptoms.

### 24-Hour dietary recall

Dietary recall will be conducted by the Automated Self-Administered 24-hour dietary assessment tool (ASA24) [48], Exploratory analysis may consider potential mediation or moderation effects of dietary intake, including total calories per carbohydrate, protein, and fat.

### Blood biomarker assessment

Capillary blood sampling using the Tasso blood collection device will be performed each test session. A trained phlebotomist will thoroughly clean the area with an alcohol swab and collect two sets of 400 uL of capillary blood sample using the Tasso device. Plasma levels of NF-L, Tau, UCH-L1, and GFAP will be assessed by Simoa 4-plex assay kits (Quanterix), as well as various exploratory markers including inflammatory cytokines and energy-related metabolites.

### Neuroimaging assessment

Neuroimaging assessments will take place in the Indiana University’s Imaging Research Facility (IRF), which houses a research-dedicated 3T Siemens Prisma MRI scanner equipped with a 64-channel head/neck coil.

High-resolution anatomical images (T1 weighted) will be acquired using 3D MPRAGE pulse sequence: TR/TE=2400/2.3ms, TI=1060ms, flip angle=8, field-of-view=256mm, matrix=320x320, bandwidth = 160 Hz/pixel, iPAT=2, resulting in 0.8 mm isotropic resolution. Two consecutive DTI sessions with opposite phase encoding directions will be performed with a simultaneous multi-slice (SMS) single-shot spin-echo echo-planar imaging (EPI) pulse sequence (TE=89.4ms; TR=3590s, 1.5mm isotropic resolution). Each session has 103 images with different diffusion weightings and gradient directions (7 b=0 s/mm^2^, 6 directions with b=500 s/mm^2^, 15 directions with b=1000 s/mm^2^, 15 directions with b=2000 s/mm^2^, and 60 directions b=2500 s/mm^2^). The DTI protocol is adapted from the Adolescent Brain Cognitive Development study,[49] which is optimized for analysis. The total scan time is 13 minutes. Resting-state data will be collected using a SMS single-shot EPI sequence with the following parameters: FOV=216mm, TR/TE=800/30ms, flip angle=52°, matrix=90×90, resolution=2.4 mm isotropic, and multiband acceleration factor=6, with 1000 total volumes acquired. Resting-state connectivity data will be acquired while the participant relaxes with eyes open and passively viewing a crosshair for 12 minutes. Two short spin-echo EPI scans with opposite phase encoding directions will be acquired for susceptibility artifact correction. Perfusion imaging will be acquired using three-dimension pulse continuous arterial spin labeling (3D-PCASL) perfusion imaging using a 3D background suppressed fast-spin-echo stack-of-spiral readout module with eight in-plane spiral interleaves (TR/TE/TI =5327ms/10.5ms/2525ms, labeling duration=1500ms, post-labeling delay=2525ms, no flow-crushing gradients, FOV=240x240mm2, in-plane matrix=128×128, number of excitation= 4, slice thickness=4mm) and an echo train length of 36 to obtain 36 consecutive axial slices [50]. Quantitative Susceptibility Mapping will be collected using a three-dimensional flow-compensated multiecho gradient-echo sequence with a repetition time of 45 msec, a total of five echoes (time of the first echo, 13msec) with echo spacing of 6 msec, flip angle=20°, FOV=24×24 cm, matrix=512×256, and 88 sections acquired with 1.5-mm thickness [51].

### Cold pressor test

The CPT procedure will take 30 min, and participants will rest quietly in the supine position the entire time. After 15 min of rest, the CPT will be performed by immersing the participant’s hand up to the wrist in agitated cold water (~0°C) for 2 min. Throughout, beat-to-beat blood pressure will be continually measured using finger photoplethysmography and heart rate using a 3-lead ECG. Data will be collected at 1,000 Hz using a data acquisition system (Powerlab), and mean values for MAP and heart rate will be extracted offline in 30 s bins. The area under the curve will then be calculated to provide an index of the cumulative sympathetic response to the CPT. Resting-state heart rate variability and spontaneous cardiovagal baroreflex sensitivity (cBRS) will be collected over the 5 min before the CPT. Using the R-R interval data collected from the ECG recordings during 5 min of paced breathing, time domain analyses will be performed to estimate overall heart rate variability and provide insight into cardiac parasympathetic activity [52]. Baroreflex sequences of 4 consecutive cardiac cycles will be captured using WinCPRS software. The mean of the regression slope will be calculated as the cBRS gain and used for analysis [53].

### Near point convergence (NPC)

NPC will be measured based on our established protocol [13–16]. Using the accommodative ruler, a target (14-point letter) will be moved toward the eyes at a rate of 1–2 cm/s. NPC will be recorded when participants report diplopia has occurred, or the tester observes eye misalignment. The assessment will be repeated twice, and the mean NPC value will be used for analyses.

### King-Devick test (KDT)

The KDT consists of a total of 145 saccades while rapidly reading numbers aloud to complete the test [54]. The KDT will be administered on a tablet. The total time (in seconds) will be used for analysis.

### Cognitive function

Cognitive function will be measured using the NIH Toolbox Cognition Battery, which has excellent reliability and validity to measure cognition in young adults [55], and has construct validity among those with TBI [56]. Multiple forms will minimize learning effects. This battery examines 5 cognitive domains (executive function, episodic memory, language, working memory, and processing speed) and takes 20 minutes to complete on a tablet [57,58].

### Ethics and dissemination

The trial will be conducted in accordance with the ethical principles outlined in the Declaration of Helsinki, 1996. This research was approved by the Indiana University Institutional Review Board (IRB #21334). Data will be collected using Research Electronic Data Capture (REDCap) spreadsheet to organize data and store in a secured database until statistical analysis is conducted. The data will be stored indefinitely for data quality purpose.

### Safety considerations and adverse events

There will be an independent safety monitor who will monitor adverse events data, oversee procedures designed to protect the privacy of participants, and report any adverse event. Data and safety monitoring will occur after every 5 participants have been enrolled. In the event of an adverse event, the safety monitor will prepare and submit a report to the IRB and will communicate between the team and IRB in any necessary investigation.

### Data analysis

#### Primary analysis.

A multivariable mixed-effect regression model (MMRM) will be used to analyze each aim separately to examine the protective effects of DHA+EPA against subconcussive neural injury. As mentioned, the primary time point is set to 24h post-heading. The models will account for repeated measures from the same participants. We expect changes in neurological outcomes (structural, physiological, autonomic impairments, cognition/N-O function) over time to differ between groups, which is reflected in the group (DHA+EPA vs. Placebo) by time (pre- vs. post-heading) interaction. Although we primarily treat time as a categorical variable (pre, 1^st^ 24h, 2^nd^ 24h, 7d, post), we will rerun the model by treating time as a continuous variable (post-heading length) as part of sensitivity analysis. All possible baseline covariates, such as demographics, omega-3 FA compositions, heading kinematics, psychiatric factors, and antioxidant levels, will be evaluated in the context of group difference.

#### Secondary analysis.

The cumulative effect from 2 heading sessions will be assessed using a MMRM by comparing 2 groups at post-heading time points, especially at 2^nd^ 24h and 168h. The role of female-related factors will be assessed by a series of MMRMs. Using a similar model structure to the primary analysis, sex (male vs. female) will be included as a term to analyze the potential group x sex interaction. We will then focus on female samples in each group to examine the potential phases of the MC (follicular vs. luteal) by group (DHA+EPA vs. placebo) interaction, and estradiol and progesterone levels will be incorporated as predictors. Lastly, the DHA+EPA group data will be stratified to assess whether there are subgroup differences [baseline DHA+EPA deficiency (<4% DHA+EPA) vs. sufficiency (≥4%)] in the outcomes, tested by the model similar to the first MMRM.

### Sample size

Based on the sample calculation for each aim, we will recruit a minimum number of subjects needed to conduct all aims. Thus, 208 participants (n=104 per primary group) will be recruited. *Alpha level setting:* The primary groups (DHA+EPA vs. placebo) share 3 post-heading time points (1^st^ 24h, 2^nd^ 24h, 168h), which will be compared to the pre-heading baseline. Thus, we set the alpha level at 0.017 to account for multiple comparisons. Based on the estimated effect sizes and alpha level, a sample size of 90 (Aim 1), 74 (Aim 2), and 82 (Aim 3) per primary group will result in 90% power to detect the supplementation effect. We accounted for a dropout rate of 15%, resulting in n=104 per group needed to accomplish all aims.

## Discussion

NF-L, tau, and S100B have been shown to reflect the severity of cellular damage ranging from acute subconcussive head impact/concussion [6,10,59] to neurogenerative diseases [60,61]. We recently showed that GFAP and UCH-L1 were elevated during a high school football season, and UCH-L1 had significant associations with the extent of brain tissue strain [62]. An interaction between omega-3 FAs and axonal integrity was suggested by Oliver et al.[31] and Heileson et al.[30] in which omega-3 FAs pretreatment in college football players minimized the elevation of NF-L, while groups given placebo continued to show elevated NF-L levels in concert with subconcussive head impact exposure throughout the season. We postulate that DHA will protect the integrity of neural cellular phospholipid membrane, by which cellular enzymatic and structural proteins remain intact even after acute soccer headings, as well as 2 sessions of cumulative subconcussive head impact exposure. If this hypothesis is supported by the blood biomarker data coupled with other measurements, this highlights a novel preventive strategy of DHA+EPA for subconcussive neural injury. It is possible that some biomarkers may increase after headings in both groups, the panel of biomarkers assessed reflect diverse aspects of neural health, such as axonal structure, neuronal protease system, and astrocyte activation. Our data analysis will determine whether DHA+EPA can act to protect all or only specific aspects of neural health.

Our pilot data [63] combined with published concussion studies [64,65] suggests that DTI and NODDI can detect both acute and long-term axonal microstructural damage. We postulate that DHA+EPA pretreatment will enhance cellular and physiological resilience to acute subconcussive impacts. Diffusion metrics may reveal microstructural integrity while the connectivity analysis depicts functional connectivity between brain regions. Using DTI/NODDI/fMRI as outcomes for omega-3 FA research, we predict significant changes in axonal diffusion measures after 20 acute headings in the placebo group and that damage will be attenuated in the DHA+EPA group. The fMRI data will indicate whether structural damage resulted in neurophysiologic abnormalities. If notable association between multimodal imaging metrics are present, the data will be useful in understanding the physiology of DHA and EPA’s protective effects.

Our data will provide mechanistic insights as to how acute subconcussive head impacts induce sympathetic hyperreactivity. A meta-analysis concluded that DHA and EPA supplementation (3-4g/day) results in a decrease in blood pressure [25], which can be modulated by both autonomic and vascular origins. DHA+EPA supplementation (3.4g/d) for 8 weeks has been shown to lower sympathetic reactivity and attenuate the MAP response during the CPT [27]. Acute subconcussive head impacts have been shown to negatively influence autonomic function by triggering sympathetic hyperreactivity. We predict that 8 weeks of DHA and EPA pretreatment will prevent sympathetic hyperreactivity and attenuate the rise in MAP during the CPT not only after a single session of soccer headings, but also after 2 sessions. If our hypothesis is confirmed, DHA and EPA have a potent protective function for the autonomic nervous system against repetitive subconcussive head impacts.

The results obtained from this study will be valuable insights into combined effect of DHA and EPA on brain molecular, cellular, and physiological health in the context of subconcussive head impacts. Furthermore, these findings will pave the way for a large-scale longitudinal randomized clinical trial, serving as empirical evidence to inform the development of preventative guidelines for athletic organizations.

### Patient and public involvement

It was not appropriate or possible to involve patients or the public in the design, or conduct, or reporting, or dissemination plans of our research.
